# Active Micro/Nanoparticles in Colloidal Microswarms

**DOI:** 10.3390/nano13101687

**Published:** 2023-05-21

**Authors:** Qianqian Wang, Dongdong Jin

**Affiliations:** 1 Jiangsu Key Laboratory for Design and Manufacture of Micro-Nano Biomedical Instruments, School of Mechanical Engineering, Southeast University, Nanjing 211000, China; 2 School of Materials Science and Engineering, Harbin Institute of Technology (Shenzhen), Shenzhen 518000, China

**Keywords:** active micro/nanoparticle, microswarm, collective behavior, micro/nanorobot, swarm control, wireless actuation

## Abstract

Colloidal microswarms have attracted increasing attention in the last decade due to their unique capabilities in various complex tasks. Thousands or even millions of tiny active agents are gathered with distinctive features and emerging behaviors, demonstrating fascinating equilibrium and non-equilibrium collective states. In recent studies, with the development of materials design, remote control strategies, and the understanding of pair interactions between building blocks, microswarms have shown advantages in manipulation and targeted delivery tasks with high adaptability and on-demand pattern transformation. This review focuses on the recent progress in active micro/nanoparticles (MNPs) in colloidal microswarms under the input of an external field, including the response of MNPs to external fields, MNP–MNP interactions, and MNP–environment interactions. A fundamental understanding of how building blocks behave in a collective system provides the foundation for designing microswarm systems with autonomy and intelligence, aiming for practical application in diverse environments. It is envisioned that colloidal microswarms will significantly impact active delivery and manipulation applications on small scales.

## 1. Introduction

In nature, swarming individuals gather together to enhance the capability of individuals and adapt to changing environments, demonstrating collective intelligence that individuals cannot achieve on their own [1,2,3,4,5]. Over the years, researchers have focused on mimicking natural collective intelligence by designing artificial and bio-hybrid swarm systems [6,7,8,9,10,11,12,13,14,15]. Among them, colloidal microswarms consisting of various building blocks have drawn increasing attention because of their fast response to external field inputs, demonstrating complex, adjustable dynamic behaviors [16,17,18,19]. Inducing the collective behavior of small-scale agents (building blocks) represents a fundamental approach to building microswarm systems. Active MNPs that exhibit a fast response to external fields are promising candidates for colloidal microswarm systems [20,21,22,23,24,25,26,27]. Adjustable particle–particle and particle–environment interactions provide diverse mechanisms for forming microswarms with on-demand collective states [28,29,30,31,32,33].

Colloidal microswarms show superior features in various manipulation and delivery tasks. Colloidal microswarms usually have a high tolerance to the size of building blocks. The tiny size of individual agents causes a limited influence on the surrounding environments and objects, whereas a swarm pattern increases the capability, such as batch operation and parallel operations [34,35,36,37]. Moreover, individual MNPs carry limited materials (e.g., drugs and contrast agents) due to the volume constraint. The delivery capability increases by inducing the collective behavior of individuals, which enables efficient delivery tasks [38,39]. Pattern navigation increases the delivery accuracy by avoiding multiple-agent tracking and control [40,41]. During manipulation or delivery tasks, the relatively fast response of colloidal microswarms enables pattern transformation, which is essential for adaptive control in complex environments [42,43,44]. Furthermore, the gathering effect enhances the imaging contrast of medical imaging, which is essential for performing targeted delivery and manipulation tasks in vivo [45,46,47,48,49].

**Figure 1:**
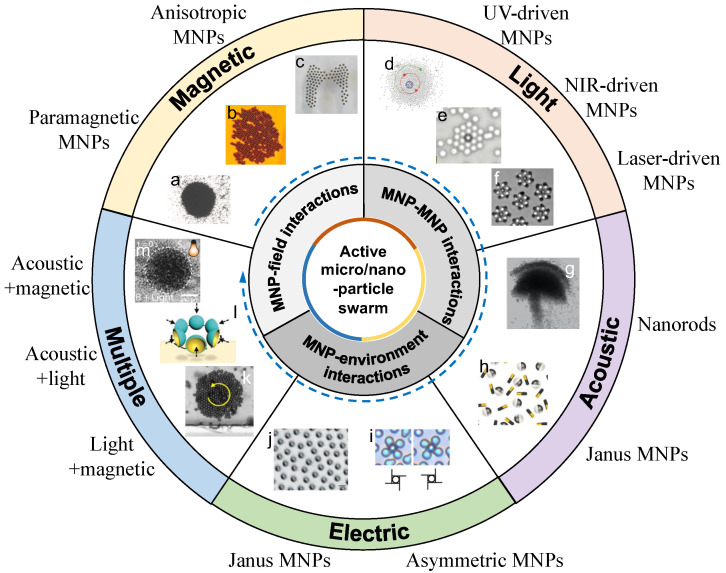
Schematic illustration of active micro/nanoparticles (MNPs) in microswarms under external fields. The insets were reproduced from the following references. Magnetic: (**a**) Adapted from [50], copyright 2018 SAGE Publications. (**b**) Reproduced from [51] under a Creative Commons CC-BY license. (**c**) Adapted from [52] under a Creative Commons CC-BY license. Light: (**d**) Adapted from [53], copyright 2014 Wiley. (**e**) Adapted from [54], copyright 2017 Wiley. (**f**) Adapted from [55], copyright 2018 Springer Nature. Acoustic: (**g**) Adapted from [56], copyright 2020 Wiley. (**h**) Adapted from [57], copyright 2015 American Chemical Society. Electric: (**i**) Adapted from [58], copyright 2015 National Academy of Sciences. (**j**) Adapted from [59], copyright 2016 Springer Nature. Multiple: (**k**) Adapted from [60] under a Creative Commons CC-BY license. (**l**) Adapted from [61], copyright 2019 Wiley. (**m**) Reproduced from [62], copyright 2020 American Chemical Society.

The design and selection of building blocks are critical steps before applying colloidal microswarms for practical applications. The response behavior of building blocks under different external inputs should be evaluated. The interaction among building blocks plays an important role in pattern control and navigation, affecting the collective interactions between building blocks and the surrounding environments. In this review, the recent progress on active MNP-based colloidal microswarms and their control methods are reviewed and discussed (Figure 1). The behaviors of active MNPs under external fields (magnetic, acoustic, and electric fields; light; and multiple fields) are summarized, including particle–particle and particle–environment interactions. The mechanisms of microswarm actuation, formation, and pattern transformation are compared and reviewed. This review intends to provide a big picture for researchers and practitioners interested in colloidal science, microrobotic control, and targeted delivery.

## 2. Active Particles in Magnetic Field-Driven Microswarms

Driven by external magnetic fields, the interactions between active magnetic colloids enable collective control and pattern formation, in which the dynamic behavior of magnetic agents is controlled by the input field (e.g., field strength and alternating frequency) and affected by the surrounding environment (e.g., viscosity and iron strength). The magnetic response of a magnetic agent is significantly connected to the magnetization curve that typically refers to ferromagnetic and paramagnetic features. The net magnetization of ferromagnetic agents (e.g., Fe, Co, and Ni particles) is dependent on the magnetization history, showing an input–output relationship between the applied field and the resulting magnetization. Paramagnetic agents disappear when removing the applied field. Compared with ferromagnetic ones, paramagnetic agents avoid remanent magnetization-induced aggregation and are widely used in targeted delivery applications. Inside a microswarm, the anisotropic features of magnetic building blocks affect the agent–agent interactions, which may provide collective behaviors that an isotropic magnetic MNP-based system finds hard to perform. To show how different building blocks affect the swarm behavior, we categorized the magnetic building blocks into three types, paramagnetic nanoparticles, paramagnetic microparticles, and anisotropic particles, based on their magnetic response under external fields.

### 2.1. Paramagnetic ${\mathrm{Fe}}_{3}O_{4}$ Nanoparticles in Microswarms

**Figure 2:**
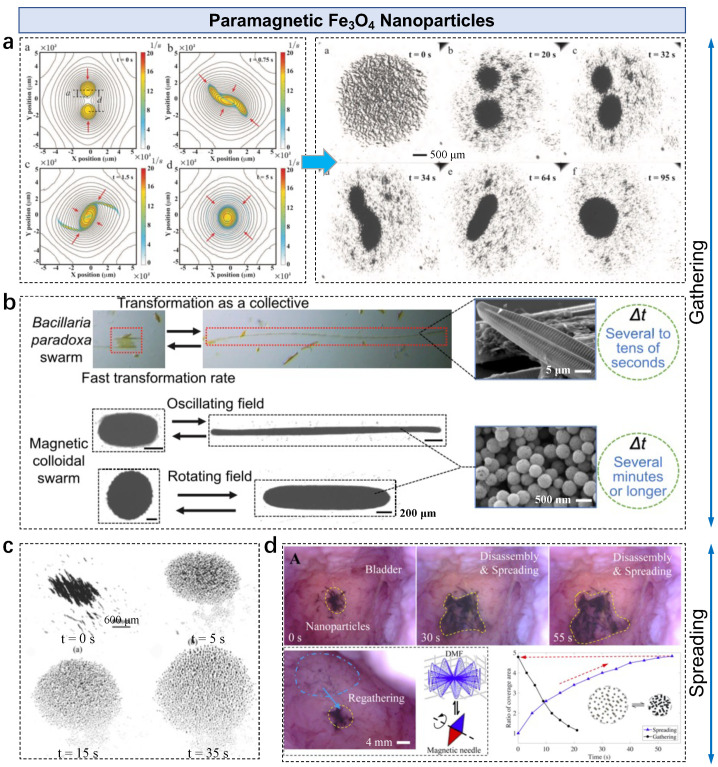
Paramagnetic ${\mathrm{Fe}}_{3}O_{4}$ nanoparticles in microswarms. (**a**) Vortex microswarm formed under a rotating magnetic field, governed by hydrodynamic interactions among nanoparticle chains. Reproduced from [50], copyright 2018 SAGE Publications. (**b**) Bio-inspired ribbon microswarms formed under an oscillating field. Reproduced from [64], copyright 2022 American Chemistry Society. (**c**) Spreading and disassembly of nanoparticle chains on a flat surface. Reproduced from [67], copyright 2017 IEEE. (**d**) Reversible spreading–disassembly and gathering of nanoparticle clusters on uneven surfaces. Reproduced from [43], copyright 2020 Elsevier.

Paramagnetic ${\mathrm{Fe}}_{3}O_{4}$ nanoparticles are widely used as building blocks for forming colloidal microswarms. Compared with general ferromagnetic materials, the hysteresis of paramagnetic MNPs is negligible. Thus, these materials can be considered as having linear magnetization within saturation [63]. Negligible hysteresis also prevents aggregation when turning off the external magnetic field, benefiting the collective control of colloidal microswarms. Driven by an in-plane rotating magnetic field, nanoparticle chains are formed by the dipole–dipole attraction among 500 nm diameter ${\mathrm{Fe}}_{3}O_{4}$ nanoparticles (Figure 2a) [50]. Each chain keeps inducing a vortex with the vorticity of

(1) $$\xi =\nabla \times u=\left(\frac{\partial u_{z}}{\partial y}-\frac{\partial u_{y}}{\partial z},\frac{\partial u_{x}}{\partial z}-\frac{\partial u_{z}}{\partial x},\frac{\partial u_{y}}{\partial x}-\frac{\partial u_{x}}{\partial y}\right)$$

where $u_{x}$, $u_{y}$, and $u_{z}$ are the three components of velocity field u. Since the vortex is two-dimensional (2D), the equation is simplified to $\xi =\left(\frac{\partial v_{y}}{\partial x}-\frac{\partial v_{x}}{\partial y}\right)\hat{z}$ considering $\xi_{z}$ is zero. The two vortices move towards each other due to the long-range attractive interaction, and the vortex merge begins when the separation distance breaks a critical value (a/d). Finally, a microswarm with a relatively high area density of nanoparticles can be observed. During the pattern formation process, both particle–particle interactions and chain–chain interactions govern the process, making the intact swarm pattern. The magnetic interactions inside a swarm pattern can be adjusted by modulating the applied field. Using the same building blocks, a ribbon-like microswarm is formed under an in-plane oscillating field ($B\left(t\right)=A\sin(2\pi ft)\hat{x}+C\hat{y}$ with *f* as the input frequency) [64,65]. Similar to the vortex-like microswarm, particle chains are first formed and exhibit oscillation motion with the field. Unlike the vortex-like microswarm governed by chain–chain hydrodynamic interactions, the ribbon-like pattern is formed by chain–chain magnetic interactions. By changing the field ratio γ (γ = A/C), the chain–chain magnetic interactions can be regulated, resulting in a pattern with an adjustable aspect ratio (Figure 2b). The pattern transformation (elongation and shrinkage) rate is significantly affected by the input field strength, frequency, and ratio. Thus, an optimized transformation rate can be defined by modeling the interactions between particle chains. A similar control method shows effectiveness on functionalized paramagnetic MNPs. Conducive ribbon-like microswarms are formed by ${\mathrm{Fe}}_{3}O_{4}$-Au core shell nanoparticles [66]. The pattern transformation capability enables on-demand bridging between electrodes and real-time switching between different electrodes.

Reversible pattern formation and spreading can be magnetically controlled. The gathered ${\mathrm{Fe}}_{3}O_{4}$ nanoparticles can disassemble or spread under a programmable field to break the inner interactions. In a low Reynolds number regime, a moving particle chain is governed by the counterbalance relationship between the driving magnetic torque and resistant drag torque. Therefore, chain fragmentation occurs by increasing the drag torque and simultaneously decreasing the magnetic torque (Figure 2c). The induced chain–chain repulsion contributes to chain spreading, resulting in the disassembly and spreading of particle chains on a flat surface [67]. Such a process is also effective on uneven surfaces or tissues by applying a 3D dynamic field $B\left(t\right)=B_{z}+B_{xy}$ [43],

(2) $$B_{z}=B\cos\theta_{m}\cdot \sin\left(2\pi f_{z}t\right)\hat{z}$$

(3) $$B_{xy}=B_{x}+B_{y}=B\sin\theta_{m}\cdot \left[\sin\left(2\pi f_{xy}t\right)\hat{x}+\cos\left(2\pi f_{xy}t\right)\hat{y}\right]$$

where $\theta_{m}$ represents the minimal precession angl and $f_{z}$ and $f_{xy}$ are the vertical and in-plane oscillating frequencies, respectively. During actuation, the relatively fast in-plane oscillation breaks long chains into short chains and the separation distance between chains is enlarged by exploiting magnetic chain–chain repulsive forces, resulting in an increased coverage area (Figure 2d). The particles can be regathered again by applying a rotating magnetic field, showing a reversible spreading–gathering process.

### 2.2. Paramagnetic Polymer Microparticles in Microswarms

**Figure 3:**
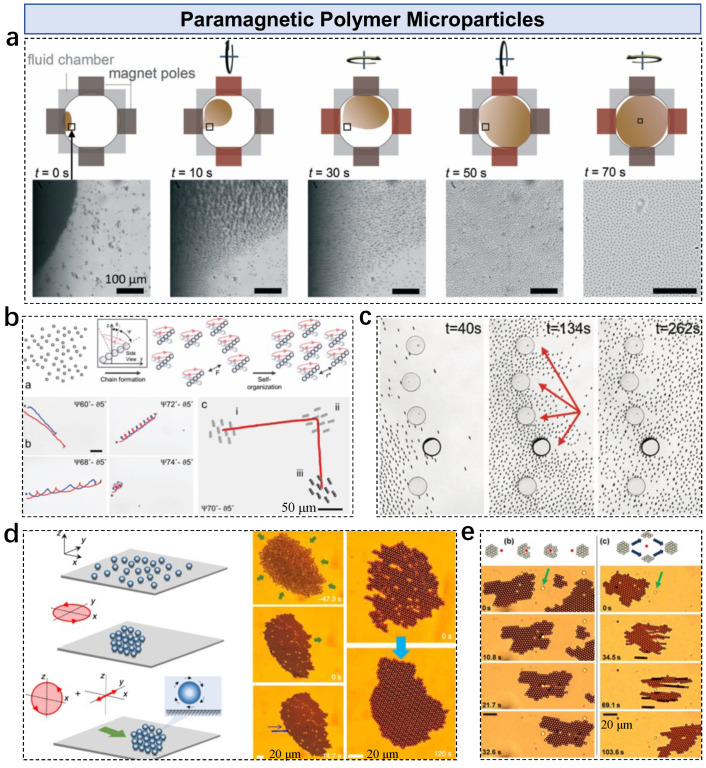
Paramagnetic polymer microparticles in microswarms. (**a**) Dispersion of dense magnetic microparticles by interfacial rotaphoresis. Reproduced from [68], copyright 2015 Royal Society of Chemistry. (**b**) Magnetic control of a microswarm under precessing magnetic fields. Adapted from [69] under a Creative Commons CC-BY license. (**c**) Microswarm navigates through an array of obstacles. Adapted from [70] under a Creative Commons CC-BY license. (**d**) Self-organization of a microswarm under dynamic magnetic fields. Adapted from [51] under a Creative Commons CC-BY license. (**e**) Microswarm moves against an immobile obstacle. Reproduced from [71], copyright 2015 American Physics Society.

Collective control of paramagnetic polymer microparticles in a swarm system has been performed with active control strategies. Unlike pure ${\mathrm{Fe}}_{3}O_{4}$ nanoparticles, these particles usually consist of iron oxide nanoparticles that are uniformly distributed inside a polymer matrix such as polystyrene. Compared with ${\mathrm{Fe}}_{3}O_{4}$ nanoparticles, polymer microparticles have a relatively insensitive magnetic response and better particle dispersion, which avoids aggregation and allow us to monitor single particle behavior in a swarm system. Dense and multilayer 2.8 μm diameter superparamagnetic particles are uniformly distributed by magnetic methods [68]. The system applies interfacial rotaphoresis consecutively in four directions, resulting in a hexagonal arrangement with a narrow distribution of particle–particle distances (Figure 3a). Pattern formation of collective microparticles relies on the balance between inner attractive and repulsive interactions [69]. Under precessing magnetic fields, cohesive clusters are formed under the balance of dipolar attractions (particle–particle interactions in a chain) and multipolar repulsions (chain–chain interactions) (Figure 3b). The inner pattern and pattern–substrate interactions are adjusted by modulating the precession and tilt angles. The microparticle swarm shows dynamic adaptation in a multiple-obstacle environment [70]. The collective chains are guided through an array of convex obstacles, which are compressed and expanded when contacting obstacles (Figure 3c). Compared to magnetic nanoparticle swarms, the disassembly of microparticle swarms allows observation of single-particle behavior in a uniformly distributed particle monolayer.

Magnetic-interaction-enabled dynamic self-assembly of microparticles plays an important role in microswarm formation. Various collective behaviors can be induced by modulating the magnetic interaction between these micro-sized building blocks. To form a carpet-like pattern of 2.8 μm diameter monodisperse paramagnetic particles, a rotating field polarized in the x-y plane is applied, as [51]

(4) $$B\left(t\right)=B_{0}\left[\cos\left(2\pi ft\right)\hat{x}-\sin\left(2\pi ft\right)\hat{y}\right]$$

where $B_{0}$ is the field strength and *f* is the frequency. To navigate the pattern, a rotating component in the x-z plane and an oscillating component along the y-axis are added, as

(5) $$B\left(t\right)=B_{0}\left[\cos\left(2\pi ft\right)\hat{x}+\sin\left(2\pi f_{y}t\right)\hat{y}-\left(B_{z}/B_{0}\right)\sin\left(2\pi ft\right)\right]$$

where $f_{y}=f/2$. The (x,z) component enables rotation-to-translation conversion, and the oscillation component maintains pattern stability (Figure 3d). Monodisperse paramagnetic particles are assembled into a magnetic carpet under a rotating field polarized in the x-z plane [71]. Interestingly, the carpet shows stability when navigating against an obstacle, i.e., an immobile microparticle (Figure 3e). The pattern is kept intact due to the relatively strong attractive dipolar interactions, in which the disordered pattern recovers after passing through the obstacle. An active strategy is designed and applied; the carpet is split before contacting the obstacles and then the pattern is reassembled, showing a dynamic reconfigurable capability. Besides the paramagnetic microparticles, ferromagnetic nickle microparticles exhibit dynamic swarm patterns at interfaces [72,73]. The active particles disturb surrounding fluids or interfaces, resulting in active swarm patterns that can be navigated by breaking the fluidic symmetry.

### 2.3. Anisotropic Magnetic Microparticles in Microswarms

**Figure 4:**
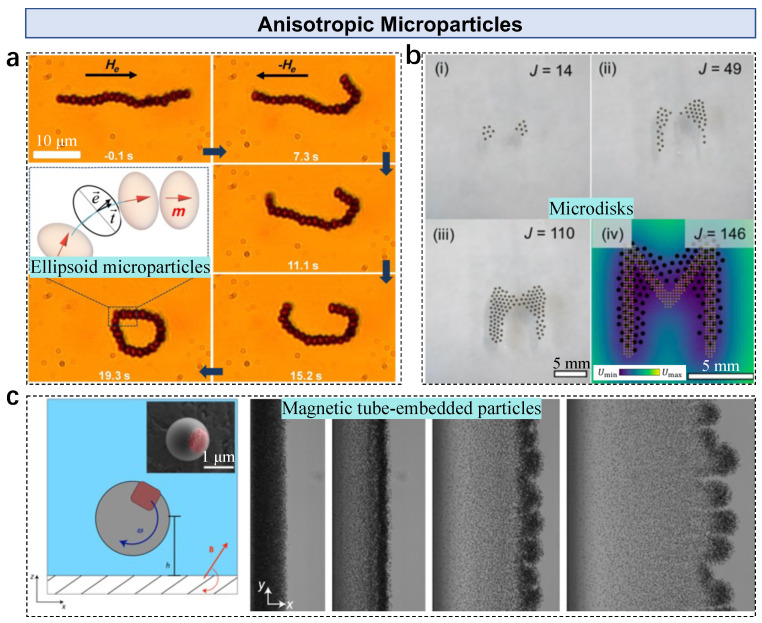
Anisotropic magnetic microparticles in microswarms. (**a**) Formation of a dipolar ring by ellipsoids. Reproduced from [75], copyright 2016 American Physics Society. (**b**) Pattern formation by magnetic microdisks. Adapted from [52] under a Creative Commons CC-BY license. (**c**) Hydrodynamic-interaction-governed microswarms by magnetic rollers. Adapted from [76], copyright 2017 Springer Nature.

Anisotropic magnetic microparticles show unique features as building blocks of microswarms due to their anisotropic structure and response to external stimuli. Thus, two types of anisotropic magnetic microparticles are categorized as shape anisotropic and magnetism anisotropic types. Peanut-shaped hematite colloidal particles (long axis: 3 μm, short axis: 2 μm) have a permanent moment along their short axis [74]. These particles aggregate into a cluster due to the residual magnetic dipole force. Unlike sphere microparticles, peanut-shaped particles show frequency-dependent responses to dynamic fields, i.e., they exhibit a rotating-to-spinning transition by increasing the input frequency of the in-plane rotating field, which is governed by the counterbalanced relationship between the driven magnetic torque and drag torque. Various collective states are demonstrated under different input fields, including the liquid state (vertical alternating field), the chain state (rotating field in the x-z plane), the vortex state (rotating field in the x-y plane), and the ribbon state (precessing field). Moreover, pattern transformation among different states can be conducted by modulating the input field, which is governed by the magnetic response of particles and the inner interactions among the particles. Taking advantage of the shape anisotropic features, dipolar rings are formed by ellipsoid microparticles [75]. In the absence of an applied field, ellipsoid microparticles (major axis: *a* = 1.8 μm, minor axis: *b* = 1.33 μm) form a ribbon due to the presence of a small moment. Adding a static field opposite to the moment allows a dipolar ring to be formed and further applied for cell caging and transportation (Figure 4a). The colloidal ring acts like a soft end-effector with deformation capabilities, benefiting cell entrapment and release.

Ferromagnetic disks with a 100–350 μm diameter and a 100 μm thickness form collective patterns at the air–water interface [52]. Active pattern transformation is performed by programming the external magnetic potential energy (Figure 4b). The anisotropic shape allows the surface tension to constrain the orientations of disks vertically and balance forces in the vertical direction. The strong inner modular repulsion among disks also prevents aggregation and stabilizes the pattern. When forming a collective pattern, multiple magnetic disks are exposed to the external field. The magnetic potential energy of the *i*th disks located at $r_{i}$ with a moment $M_{i}$ is a superposition of the potential energy resulting from both ($B_{ext}$) and inter-modular ($B_{int}$) fields, as

(6) $$U_{i}=U^{inter}\left(M_{i},B_{int}\right)+U^{ext}\left(M_{i},B_{ext}\right)$$

During actuation, all disks move towards a position where a local or global minimum of the external magnetic potential energy exists. Therefore, by programming the external potential energy (distribution of permanent magnets), the collective pattern can be dynamically changed to the desired shape or exhibit controlled pattern transformation. Besides the shape anisotropic magnetic building blocks, Driscoll et al. used polymer microparticles with an embedded magnetic cube as building blocks [76]. Under an x-y plane rotating magnetic field, motile structures are formed by hydrodynamic interactions, showing fingering instability (Figure 4c). Autonomous ‘critters’ can form when fingers pinch off from an unstable front, which move faster than individual particles.

## 3. Active Particles in Light-Driven Microswarms

Radiation energy from light is suitable for driving photoresponsive agents in a wireless and remote manner. In a microswarm system, photoactive materials are widely applied to induce inner interactions between building blocks, such as the solute concentration gradient around AgCl and ${\mathrm{TiO}}_{2}$ particles and various Janus particles (e.g., ${\mathrm{TiO}}_{2}/\mathrm{Pt}$ and ${\mathrm{TiO}}_{2}/\mathrm{Au}$ Janus particles). A precisely tuned energy input or a reversible on/off state of radiation offers on-demand control of photoactive agents and the inner pattern interactions.

Self-diffusiophoresis-governed silver chloride microparticles show collective behavior under ultraviolet (UV) light irradiation [77]. As a result of asymmetric photodecomposition, these AgCl particles move at speeds of up to 100 μm/s under UV light. The reaction (pH = 5) is expressed as

(7) $$4\mathrm{AgCl}+2H_{2}O\overset{hv,{\mathrm{Ag}}^{+}}{⟶}4\mathrm{Ag}+4H^{+}+4{\mathrm{Cl}}^{-}+O_{2}$$

**Figure 5:**
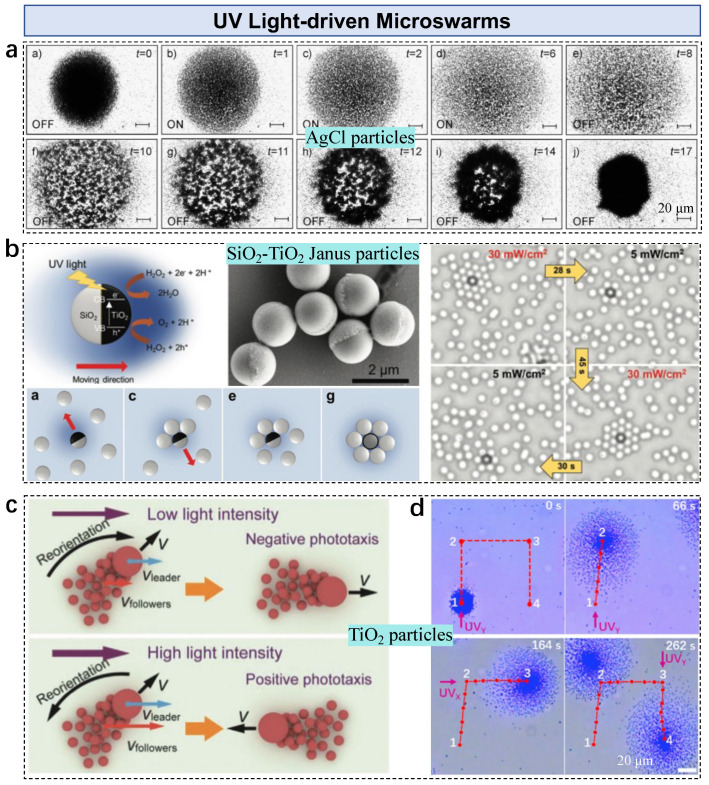
UV light-driven particles in microswarms. (**a**) AgCl particles exposed to on–off-controlled UV light. Adapted from [77], copyright 2009 Wiley. (**b**) Assembly of UV light-activated self-propelled Janus particles. Adapted from [54], copyright 2017 Wiley. (**c**) Reversible collective behavior of ${\mathrm{TiO}}_{2}$ microparticles under different light intensities. Adapted from [78], copyright 2020 Wiley. (**d**) Navigation of a ${\mathrm{TiO}}_{2}$ microswarm under UV light. Adapted from [79] under the Creative Commons CC-BY-NC-ND license.

The production of $H^{+}$ and ${Cl}^{-}$ induces an electrolyte gradient, resulting in particle motion. These particles then gather, where the short-range repulsive electrostatic interactions prevent physical contact. Switching between UV and visible light allows a tighter pattern to be observed under UV light, and then re-expansion can be observed under visible light (Figure 5a). Besides light-induced self-diffusiophoresis, UV-driven Janus microparticles also exhibit motion in solutions. Under UV irradiation, ${\mathrm{SiO}}_{2}$-${\mathrm{TiO}}_{2}$ microparticles in $H_{2}O_{2}$ solution exhibit motion due to the photocatalytic decomposition of $H_{2}O_{2}$ at the ${\mathrm{TiO}}_{2}$ hemisphere [54]. The reaction-induced chemical gradient leads to self-phoretic motion towards the ${\mathrm{TiO}}_{2}$ half. Under UV illumination, dynamic self-assembly of active (Janus particles) and passive (${\mathrm{SiO}}_{2}$ particles) particles occurs (Figure 5b). When switching the light, the clusters melt as Brownian diffusion takes over. The UV intensity affects the assembly behavior. The active particle attracts two or more shells of passive particles when increasing the intensity by 5–30 mW/cm^2^. The outer shell diffuses when reducing the intensity to 5 mW/cm^2^ because of the insufficient interactions between the passive and active particles.

Microswarms of building blocks of different sizes show interesting behavior due to different responses under light irradiation. Microswarms formed by large (diameter: 2 μm) and small (diameter: 0.5 μm) ${\mathrm{TiO}}_{2}$ particles show a switchable phototaxis under different UV light intensities [78]. Both positive and negative phototaxes are demonstrated by adjusting the UV intensity to 500 mW/cm^2^ and 4 mW/cm^2^, respectively (Figure 5c). The negative phototaxis is caused by non-electrolyte self-diffusiophoresis, i.e., the larger particle becomes the pattern leader due to the higher phototactic velocity. Navigation of ${\mathrm{TiO}}_{2}$ microswarms can be achieved using on–off recycling UV light [79]. ${\mathrm{TiO}}_{2}$ particles move toward the side-applied UV source in a phototactic manner, demonstrating a spreading state. The motion recovers to random Brownian motion without UV light, and particles regather into a circular pattern. Thus, swarm navigation is performed by applying an on–off repeating UV source to trigger the spreading–regathering states, enabling pattern locomotion (Figure 5d).

**Figure 6:**
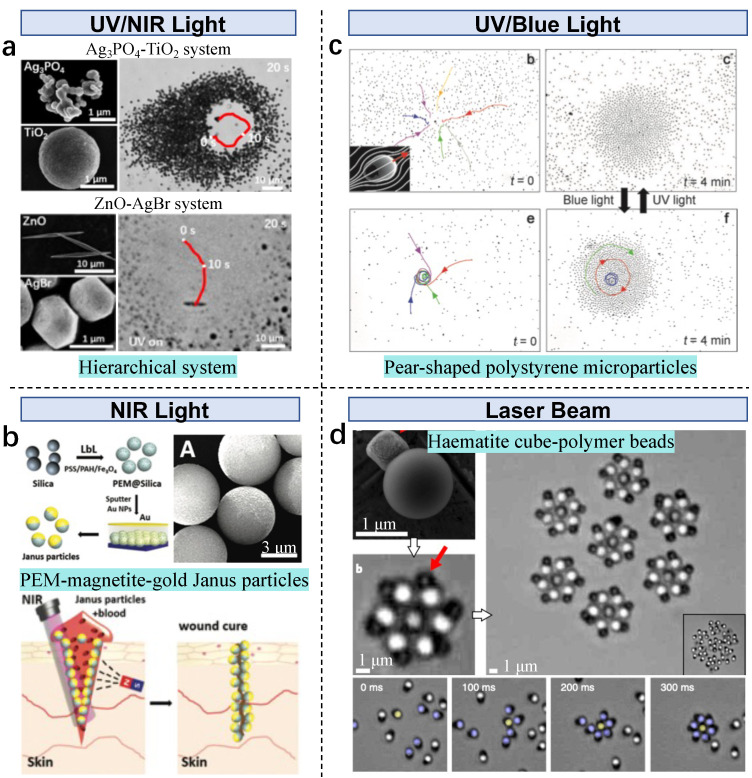
Light-driven active particles in microswarms. (**a**) Prey and predator particles in light-driven hierarchical microswarms. Reproduced from [80], copyright 2019 American Chemical Society. (**b**) Janus particles under NIR light for tissue welding. Adapted from [81] under the Creative Commons CC-BY license. (**c**) Pear-shaped polystyrene microparticles in UV/blue light-driven microswarms. Reproduced from [53], copyright 2014 Wiley. (**d**) Haematite cube polymer beads in a laser-driven microswarm. Adapted from [55], copyright 2018 Springer Nature.

Besides homogeneous building block systems, multiple types of MNPs in a heterogeneous collective system demonstrate new behaviors that homogeneous systems are unable to perform. A binary particle system consisting of two types of building blocks is proposed: diffusiophoretic attractive microparticles and diffusiophoretic repulsive particles [80]. Various collective systems can be created if binary repulsive–attractive diffusiophoretic interactions are introduced in heterospecific particles (Figure 6a). ZnO and ${\mathrm{Ag}}_{3}{\mathrm{PO}}_{4}$ particles release low-diffusivity cations and high-diffusivity anions in water

(8) $$\mathrm{ZnO}+H_{2}O\to {\mathrm{Zn}}^{2+}+2{\mathrm{OH}}^{-}$$

(9) $${\mathrm{Ag}}_{3}{\mathrm{PO}}_{4}+H_{2}O⇋3{\mathrm{Ag}}^{+}+{\mathrm{OH}}^{-}+{\mathrm{HPO}}_{4}^{2-}$$

In the ${\mathrm{Ag}}_{3}{\mathrm{PO}}_{4}-{\mathrm{TiO}}_{2}$ system, the ions create a diverging electric field, resulting in a pumping effect around ${\mathrm{Ag}}_{3}{\mathrm{PO}}_{4}$ microparticles adjacent to the ${\mathrm{TiO}}_{2}$ microparticles, demonstrating a chasing–escaping behavior. Under UV irradiation, AgBr microparticles produce a diffusiophoretic attraction to ZnO microparticles by producing high-diffusivity cations and low-diffusivity anions, showing a chasing–escaping behavior as well. PEM–magnetite–Au thermophoretic Janus particles generate heat under near-infrared rays (NIR), which is applied for assisted tissue welding [81] (Figure 6b).

Functionalized substrates enable new behavior of light-driven microswarms. Pear-shaped polystyrene microparticles (3 × 4 μm) on a photosensitive azosilane monolayer exhibit unique behavior [53]. The layer adopts cis–trans reversion when switching UV to blue light. Swarming particles show aster-like and vortex-like patterns under an alternating electric field, and pattern transition is achieved by switching the UV irradiation to blue light (Figure 6c). Haematite (α-${\mathrm{Fe}}_{2}O_{3}$) cube-bonded polymer beads decompose hydrogen peroxide under a laser beam and form dynamical superstructures in an autonomous manner [55] (Figure 6d). The formed gears are coupled through the generated chemical clouds by diffusiophoresis, and they constitute the elementary bricks of synchronized superstructures that autonomously regulate the dynamics. The amplitude of the phoretic coupling from the radial repulsion is expressed as

(10) $$\varepsilon =\frac{\alpha }{R^{3}}{\left(\frac{R}{r-R}\right)}^{8}∼2{\left(\frac{R}{r-R}\right)}^{8}$$

where *r* is the center-to-center distance between the particles and *R* is the particle radius (3 μm). Such light-driven self-propelled microswarms show the interplay between phase synchronization and spatial organization, indicating a potential new approach to forming dynamical superstructures.

## 4. Active Particles in Acoustic-Field-Driven Microswarms

Acoustic waves as a powerful driving source have been widely applied in noncontact actuation and micromanipulation tasks where tiny agents can be wirelessly controlled by modulating the acoustic wave, e.g., modulating its amplitude and frequency. Colloidal building blocks are subjected to an acoustic radiation force that is usually caused by piezoelectric-transducer-generated waves. Thus, unlike light-driven microswarms that require the forming agents to have certain responses to light and suitable chemical environments, the application range of acoustic-field-driven microswarms is wider. However, particles with a size smaller than the wavelength are hard to control since the acoustic force is proportional to the object’s size [82].

**Figure 7:**
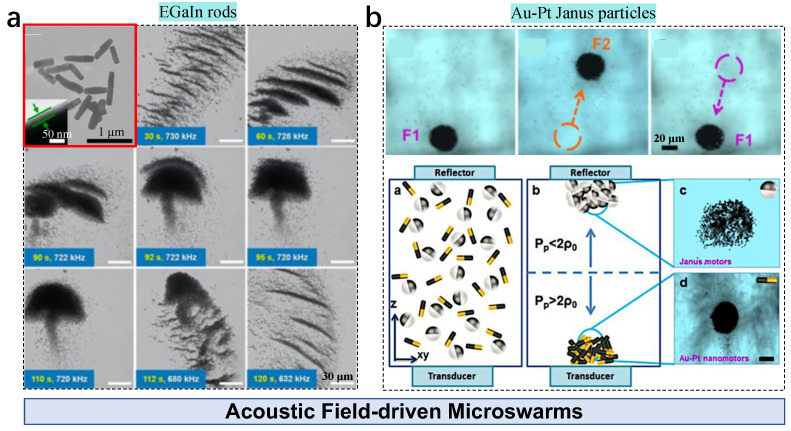
Acoustic-field-driven active particles in microswarms. (**a**) Formation of EGaIn nanorod microswarms under an acoustic field. Adapted from [56], copyright 2020 Wiley. (**b**) Formation and navigation of Au-Pt Janus microswarms under an acoustic field. Adapted from [57], copyright 2015 American Chemical Society.

Microswarms of liquid metal agents are formed under acoustic fields [56]. With an input frequency of 730 kHz, stripes of EGaIn nanorods are assembled and merge into larger stripes by reducing the frequency to 728 kHz (Figure 7a). A dandelion-like pattern is observed by reducing the frequency to 720 kHz, which is dispersed by decreasing the frequency below 680 kHz. During the assembly–disassembly process, the pattern of sound pressure changes with the frequency, i.e., the standing-wave-induced primary acoustic radiation forces change with the input parameters. These nanorods are subjected to force along the acoustic energy gradient, causing different motion states.

Besides pattern state control, collective navigation can also be performed by modulating the input acoustic source, as demonstrated by a swarm of Pt-Au nanowires (diameter: 200 nm, length 2 μm) in a $H_{2}O_{2}$ solution [57]. Without an acoustic field, the nanowires exhibit electrophoretic-propulsion-induced spreading with autonomous motion. The acoustically generated standing-wave-induced gradient drives the pattern assembly towards the low-pressure region, resulting in a region of dense nanowires. Pattern locomotion is implemented by changing the position of pressure nodes via adjusting the acoustic frequency (Figure 7b). The pattern moves with an intact state at a velocity of around 45 μm/s. In this system, the acoustic force is divided into primary and secondary radiation forces. The secondary radiation force is generated by the acoustic waves rescattered by the nanowires. It increases by decreasing the agent–agent distance [83]. Doinikov’s theory indicates that agent separation can be achieved by a radiation force (*F*) in a plane standing wave if the sphere radius ($R_{0}$) is smaller than the penetration depth of the viscous ($\delta_{v}$) and thermal ($\delta_{t}$) wave, as

(11) $$F=\pi \rho_{0}{\left|A\right|}^{2}\sin\left(2kd\right){\left(kR_{0}\right)}^{3}\left[\frac{2\rho_{0}-\rho_{p}}{3\rho_{0}}+O\left(\frac{R_{0}}{\delta_{v}},\frac{R_{0}}{\delta_{t}}\right)\right]$$

where $\rho_{o}$ is the fluid density, *A* is the complex amplitude of the velocity potential of the imposed sound field, *d* is the distance between the sphere’s equilibrium center and the wave’s velocity node plane, k=ω/c is the wavenumber, and *c* is the sound speed in the fluid. Considering a system with Au-Pt Janus nanowires and sphere particles (diameter: 1.21 μm), the density of the Janus nanowires is much larger than that of the fluid ($\rho_{p}>\rho_{0}$), leading to F<0. Thus, these nanowires migrate towards the pressure antinodes at the bottom (Figure 7b). *F* becomes positive because of the relatively low density of the Janus particles ($\rho_{p}<2\rho_{0}$); therefore, particles move toward pressure nodes located at the cell’s top. Finally, agent separation is achieved.

## 5. Active Particles in Electric-Field-Driven Microswarms

**Figure 8:**
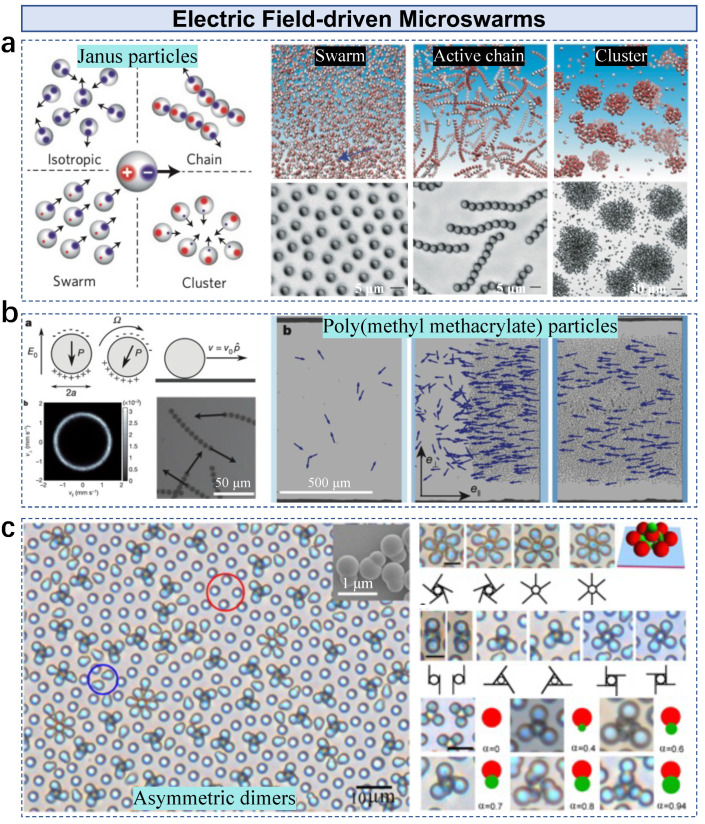
Electric-field-driven active particles in microswarms. (**a**) Janus colloidal spheres in microswarms with imbalanced, off-centered charges. Adapted from [59], copyright 2016 Springer Nature. (**b**) Collective motion of rolling particles. Adapted from [84], copyright 2013 Springer Nature. (**c**) Asymmetric dimers in a collective system. Adapted from [58], copyright 2015 National Academy of Sciences.

Under the influence of electric fields, inner attraction or repulsion between building agents is mainly determined by their charges and distance. Electrostatic imbalance provides fundamental mechanisms for forming electric-field-driven microswarms. Induced imbalanced interactions reconfigure active particles into various collective states [59]. Janus silica microparticles with one hemisphere coated with metal are the building blocks; thus, the local-field-induced ionic flows have different magnitudes on the two hemispheres. The collective state is able to be tuned by modulating the input field frequency. These particles exhibit self-propulsion in a gas state, driven by charge-induced electrophoresis (Figure 8a). Particle–particle strong ionic screening effects and negligible dipolar interactions result in isotropic movement. By increasing the input frequency, the dipolar interactions increase and repulsive interactions between metallic hemispheres dominate the swarm state. The generated attraction by the two dipoles (red and blue in Figure 8a) yields an active chain state with a further increased input frequency. The clustering of particles is performed by adding salt to the medium to tune the dipolar interactions.

The area fraction of building blocks determines the collective state of electric-field-driven microswarms. Electrostatic torque drives polymer particles to roll in random directions [84]. An isotropic gas phase is observed at a low area fraction, where all particles exhibit random rotation at the same speed (Figure 8b). A macroscopic band generates and propagates at a constant velocity through an isotropic phase when the area fraction is above a critical value. The band is independent of the field amplitude and the particle’s motion velocity. A homogeneous polar liquid phase is observed by increasing the particle fraction, where the head of the propagating bands catches up with their own tail, demonstrating that hydrodynamic interactions can drive individual particles into collective motion. Polarization of asymmetric particles leads to various collective behaviors under electric fields. Ma et al. used polystyrene dimers with asymmetric lobes as the building blocks [58]. These colloidal dimers were actuated by an alternating field between the top and bottom ITO glasses. Both chiral and achiral structures are assembled by dimers with different geometric ratios, governed by the in-plane dipolar repulsion between petals and out-of-plane attraction between the central dimer and the surrounding petals (Figure 8c). The assembled structures depend on the number of dimers involved and the radius ratio. For example, all structures with four or fewer petals are chiral, whereas achiral structures are assembled with more than five dimers. The chirality disappears if the ratio of the radii approaches zero or one.

## 6. Active Particles in Multiple-Field-Driven Microswarms

**Figure 9:**
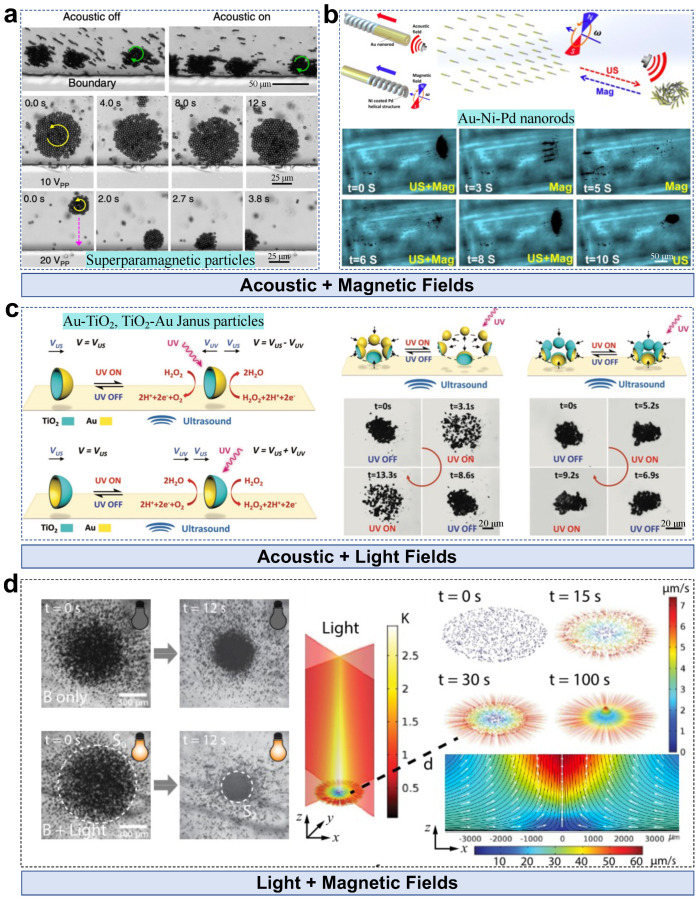
Multiple-field-actuated particles in microswarms. (**a**) Rotating acoustic–magnetic microparticle microswarms near a boundary. Adapted from [60] under a Creative Commons CC-BY license. (**b**) Collective behavior of magneto–acoustic nanorods. Adapted from [85], copyright 2015 American Chemical Society. (**c**) Collective behavior of Janus microbowls under light modulation and magnetic actuation. Adapted from [61], copyright 2019 Wiley. (**d**) Hovering ${\mathrm{Fe}}_{3}O_{4}@{\mathrm{SiO}}_{2}$ nanoparticle microswarms. Reproduced from [62], copyright 2020 American Chemical Society.

MNPs powered by multiple external fields allow microswarm control with more degrees of freedom, offering diverse collective behaviors that single energy-driven swarm systems are unable to perform. Superparamagnetic microparticles form a rotating microswarm under a rotating magnetic field [60]. The acoustic radiation force exerted on the pattern keeps the pattern in contact with the boundary, in which rotation-to-translation occurs for pattern navigation (Figure 9a). The average force on the particle (radius: *a*, a<<λ; density: $\rho_{s}$) in a standing wave of wavenumber $k_{y}$ and pressure field $p=p_{a}\cos\left(k_{y}y\right)\cos\left(\omega t\right)$ is expressed as

(12) $$F_{R}=4\pi \phi \left(\tilde{\kappa },\tilde{\rho }\right)k_{y}a^{3}E_{a}\sin\left(2k_{y}y\right)$$

(13) $$\phi \left(\tilde{\kappa },\tilde{\rho }\right)=\frac{1}{3}\left[\frac{5\tilde{\rho }-2}{2\tilde{\rho }+1}-\tilde{\kappa }\right]$$

(14) $$\tilde{\rho }=\frac{\rho_{s}}{\rho_{0}},\tilde{\kappa }=\frac{\kappa_{s}}{\kappa_{0}}$$

where Φ is the acoustophoretic contrast factor, $\rho_{o}$ is the liquid density, $\kappa_{0}$ and $\kappa_{s}$ are the compressibilities of water and the particle, respectively, $p_{a}$ denotes the pressure amplitude, and $E_{a}$ denotes the acoustic energy. Based on this calculation, the particles are forced to move towards the boundary. A relatively smaller acoustic force is insufficient to induce a pattern–boundary interaction, which leads to drifting motion. A higher translational velocity is observed by enhancing the pattern–boundary interaction by increasing the input voltage and the rotating frequency, demonstrating a combined control effect. Helical nanorods with concave ends are applied as the building blocks in a magneto-acoustic microswarm system [85]. The collective behaviors of the nanorod swarm can be switched between aggregation, swarm motion, and swarm vortexes by controlling the field input, in which the relationship between magnetic and acoustic interactions governs the collective state (Figure 9b). By turning on the two fields, a vortex-like pattern is observed. It is disassembled into a chain state with only the magnetic field input. Applying the acoustic field again leads to pattern reformation at the original node position. A stable pattern is formed by removing the magnetic field, showing a reversible, controllable collective state.

Besides the acoustic–magnetic combined control input, an acoustic field together with light input has been applied for controlling a swarm of Janus microbowls [61]. Two types of Janus microbowls are involved in the system: ${\mathrm{TiO}}_{2}$-Au and Au-${\mathrm{TiO}}_{2}$ microbowls with Au and ${\mathrm{TiO}}_{2}$ exterior layers, respectively. Under an acoustic field, both ${\mathrm{TiO}}_{2}$-Au and Au-${\mathrm{TiO}}_{2}$ microbowls exhibit motion towards their exterior side. This is caused by the oscillating-edge-generated second-order acoustic streaming flow [86], which explains why both ${\mathrm{TiO}}_{2}$-Au and Au-${\mathrm{TiO}}_{2}$ microbowls show similar collective gathering behaviors, i.e., microbowls gather with the exterior side facing the pattern center (Figure 9c). In contrast, light-induced prophetic motion is material dependent. Janus microbowls act like electrochemical cells in $H_{2}O_{2}$:

(15) $${\mathrm{TiO}}_{2}+hvs.\to {\mathrm{TiO}}_{2}\left(e^{-}+h^{+}\right)$$

(16) $$H_{2}O_{2}+2\;h^{+}\overset{{\mathrm{TiO}}_{2}}{⟶}2H^{+}+O_{2}$$

(17) $$H_{2}O_{2}+2H^{+}+2e^{-}\overset{\mathrm{Au}}{⟶}2H_{2}O$$

Therefore, Janus microbowls show pattern expansion or contraction depending on the materials of the exterior layers (Figure 9c).

Light–magnetic combined control of collective magnetic polymer particles is demonstrated in [87]. Most of the antiferromagnetic hematite cube is encapsulated by 3- methacryloxypropyl trimethoxysilane (TPM) with some parts exposed to hydrogen peroxide solution. The $H_{2}O_{2}$ decomposition-generated chemical concentration gradients, together with osmotic and phoretic effects, are controlled under blue light illumination, and crystalline structures are gradually observed. They are disassembled after turning off the light due to thermal diffusion, whereas a static magnetic field can suppress the rotational diffusion because of magnetic alignment. Under light–magnetic combined actuation, controlled locomotion with a stable pattern is achieved. However, pattern formation may fail if the initial particle–particle distance exceeds a critical value, yielding a loosely interacting system. Besides swarm control in a 2D plane, magnetic–light combined control enables hovering of paramagnetic ${\mathrm{Fe}}_{3}O_{4}@{\mathrm{SiO}}_{2}$ nanoparticles in a 3D space [62]. A 3D tornado-like microswarm is realized by integrating the magnetically induced vortex and light-induced convection flow under the combined control of a precessing magnetic field and vertical light irradiation (Figure 9d). The control strategy enables vertical growth and vertical mass transportation light irradiation, which is applied to accelerate chemical reactions.

## 7. Outlook

Microswarms consisting of various building blocks have been proposed over the past decade and are drawing increasing attention in different scientific fields. The behavior and actuation state of a microswarm are highly affected by the intrinsic properties of the building blocks. First, the response time and behavior of the building blocks under external fields determine whether they are suitable for manipulation in specific environments, such as viscous bio-fluids and solutions with a high ionic strength [88,89,90,91,92,93]. Second, the pair interactions between building blocks affect the pattern state, in which emerging collective behaviors are possibly induced, especially in an out-of-equilibrium system. Active pattern transformation can be conducted by regulating the pair interactions, providing mechanisms for adaptive control in confined, complex environments [94]. The interaction between building blocks and environment provides fundamental mechanisms for swarm navigation, since most swarm navigation requires the involvement of the boundary. Multiple-field-controlled colloidal microswarms represent a research opportunity. The recent progress we discussed shows that the multiple responses of building blocks under multiple fields offer more possibilities for pattern formation, state transformation, and on-demand morphology control, which enhances the capabilities of swarm delivery and increases the potential for achieving tasks that single field control is unable to perform [95,96]. Multiple-field-controlled colloidal microswarms usually require a relatively complex control strategy since the coupling effect may bring negative effects to the swarm system. Thus, a systematic control system design is required before applying these microswarms in complex tasks. Table 1 summarizes and compares the controllability and typical applications of active colloidal microswarms.

The group-level functionality of microswarms could be enhanced by involving heterogeneous colloidal building blocks. Several key factors need consideration during system design. First, the types of building blocks and the proportion ratio should be evaluated to meet the functionality requirements, such as imaging agents and drug carriers. The relationships among different components from a material and a physics aspect are critical for the heterogeneous system, in which a division-of-labor manner is enhanced by linking the unique features of different components [102]. Second, the pair interactions require calculation or modeling to ensure a successful collective state can be acquired, especially for a system under multiple field control. Third, a real-time control strategy is necessary to achieve synchronous operation of collective patterns in a coordinated manner. An adaptive microswarm with cooperative functions encoded will meet multitasking requirements with superior collective intelligence [103,104,105,106,107,108]. As an interdisciplinary research field, colloidal microswarm systems require intense research efforts and close collaboration between different fields, including materials, physics, control, robotics, and biomedical engineering. In the following decades, active MNPs with better controllability, biocompatibility, and biodegradability will revolutionize targeted delivery and micromanipulation, and we believe research opportunities exist in bringing collective intelligence to these fields.

## 8. Summary

In summary, we discuss the recent progress in active MNPs in colloidal microswarms that can perform on-demand collective behavior under different power inputs, including magnetic fields, light, acoustic fields, electric fields, and multiple combined fields. The response of MNPs under different energy inputs is first discussed, which provides a fundamental understanding of the MNP–field interactions and MNP–MNP interactions. The state control and motion control of microswarm patterns are then summarized and compared, highlighting the effect of MNPs on the corresponding collective control strategy. Moreover, some of the representative applications are summarized and compared to demonstrate the connection between swarm control and practical applications. Finally, the outlook and perspective of active MNP-based colloidal microswarms are given from the aspects of a fundamental understanding and practical applications.

## Figures and Tables

**Table 1:** Summary and Comparison of Controllability and Typical Applications of Active Colloidal Microswarms.

Driven Power	Controllability	Typical Examples of Applications	Ref
Magnetic field	Adjusting the pattern transformation by modulating the input field;	Pattern-transformation-enabled circuit repair using conductive nanoparticles	[66]
	Collective navigation via pattern–environment interactions	Collective manipulation of a relatively large object	[74]
		Controlled magnetic hyperthermia	[97]
Light	Pattern formation via light-excited interactions;	Tumor treatment under NIR	[98]
	Collective navigation by controlling the light source or phototaxis	Triple negative breast cancer treatment under NIR	[99]
		Colon cancer treatment under NIR	[100]
Acoustic field	Collective formation acoustic at the nodal plane; Reversible pattern control by modulating the input frequency	Collective manipulation	[57,101]
Electric field	Dynamic pattern control under AC electric fields; State transition by modualting building block’s concentration	Collective manipulation	[59,84]
Multiple powers	Offering more possibilities for collective behavior;	On-demand collective formation and motion control	[87]
	Enabling on-demand pattern transformation and adaptive control	Collective navigation in three-dimensional space	[60]
		Active pattern formation	[44]

## References

[1] Anderson C., Theraulaz G., Deneubourg J.L. (2002). Self-Assemblages in Insect Societies. Insectes Sociaux.

[2] Vicsek T., Zafeiris A. (2012). Collective Motion. Phys. Rep..

[3] Peleg O., Peters J.M., Salcedo M.K., Mahadevan L. (2018). Collective Mechanical Adaptation of Honeybee Swarms. Nat. Phys..

[4] Mlot N.J., Tovey C.A., Hu D.L. (2011). Fire Ants Self-Assemble into Waterproof Rafts to Survive Floods. Proc. Natl. Acad. Sci. USA.

[5] Peeters C., De Greef S. (2015). Predation on Large Millipedes and Self-Assembling Chains in Leptogenys Ants from Cambodia. Insectes Sociaux.

[6] Feinerman O., Pinkoviezky I., Gelblum A., Fonio E., Gov N.S. (2018). The physics of cooperative transport in groups of ants. Nat. Phys..

[7] Vach P.J., Walker D., Fischer P., Fratzl P., Faivre D. (2017). Pattern Formation and Collective Effects in Populations of Magnetic Microswimmers. J. Phys. D Appl. Phys..

[8] Wu Z., Troll J., Jeong H.H., Wei Q., Stang M., Ziemssen F., Wang Z., Dong M., Schnichels S., Qiu T. (2018). A Swarm of Slippery Micropropellers Penetrates the Vitreous Body of the Eye. Sci. Adv..

[9] Wang Q., Zhang L. (2021). External power-driven microrobotic swarm: From fundamental understanding to imaging-guided delivery. ACS Nano.

[10] Servant A., Qiu F., Mazza M., Kostarelos K., Nelson B.J. (2015). Controlled *Vivo* Swim. A Swarm Bact.-Like Microrobotic Flagella. Adv. Mater..

[11] Yan X., Zhou Q., Vincent M., Deng Y., Yu J., Xu J., Xu T., Tang T., Bian L., Wang Y.X. (2017). Multifunctional Biohybrid Magnetite Microrobots for Imaging-Guided Therapy. Sci. Robot..

[12] Li J., Dekanovsky L., Khezri B., Wu B., Zhou H., Sofer Z. (2022). Biohybrid Micro-and Nanorobots for Intelligent Drug Delivery. Cyborg Bionic Syst..

[13] Loghin D., Tremblay C., Mohammadi M., Martel S. (2017). Exploiting the Responses of Magnetotactic Bacteria Robotic Agents to Enhance Displacement Control and Swarm Formation for Drug Delivery Platforms. Int. J. Robot. Res..

[14] Felfoul O., Mohammadi M., Taherkhani S., De Lanauze D., Xu Y.Z., Loghin D., Essa S., Jancik S., Houle D., Lafleur M. (2016). Magneto-Aerotactic Bacteria Deliver Drug-Containing Nanoliposomes to Tumour Hypoxic Regions. Nat. Nanotechnol..

[15] Xu H., Medina-Sánchez M., Maitz M.F., Werner C., Schmidt O.G. (2020). Sperm Micromotors for Cargo Delivery through Flowing Blood. ACS Nano.

[16] Petrichenko O., Kitenbergs G., Brics M., Dubois E., Perzynski R., Cēbers A. (2020). Swarming of Micron-Sized Hematite Cubes in a Rotating Magnetic Field—Experiments. J. Magn. Magn. Mater..

[17] Wang Q., Yang L., Yu J., Vong C.I., Chiu P.W.Y., Zhang L. Magnetic Navigation of a Rotating Colloidal Swarm Using Ultrasound Images. Proceedings of the IEEE/RSJ International Conference on Intelligent Robots and Systems.

[18] Hwang G., Paula A.J., Hunter E.E., Liu Y., Babeer A., Karabucak B., Stebe K., Kumar V., Steager E., Koo H. (2019). Catalytic Antimicrobial Robots for Biofilm Eradication. Sci. Robot..

[19] Wang Q., Wang B., Yu J., Schweizer K., Nelson B.J., Zhang L. Reconfigurable Magnetic Microswarm for Thrombolysis under Ultrasound Imaging. Proceedings of the IEEE International Conference on Robotics and Automation.

[20] Doostmohammadi A., Ignés-Mullol J., Yeomans J.M., Sagués F. (2018). Active nematics. Nat. Commun..

[21] Zhang J., Alert R., Yan J., Wingreen N.S., Granick S. (2021). Active phase separation by turning towards regions of higher density. Nat. Phys..

[22] Han K., Kokot G., Das S., Winkler R.G., Gompper G., Snezhko A. (2020). Reconfigurable structure and tunable transport in synchronized active spinner materials. Sci. Adv..

[23] Liu J., Wang H., Liu M., Zhao R., Zhao Y., Sun T., Shi Q. (2022). POMDP-Based Real-Time Path Planning for Manipulation of Multiple Microparticles via Optoelectronic Tweezers. Cyborg Bionic Syst..

[24] Nacev A., Weinberg I., Stepanov P., Kupfer S., Mair L., Urdaneta M., Shimoji M., Fricke S., Shapiro B. (2015). Dynamic Inversion Enables External Magnets to Concentrate Ferromagnetic Rods to a Central Target. Nano Lett..

[25] Deng Z., Mou F., Tang S., Xu L., Luo M., Guan J. (2018). Swarming and Collective Migration of Micromotors under Near Infrared Light. Appl. Mater. Today.

[26] Yang M., Guo X., Mou F., Guan J. (2022). Lighting up Micro-/Nanorobots with Fluorescence. Chem. Rev..

[27] Soto F., Karshalev E., Zhang F., Esteban Fernandez de Avila B., Nourhani A., Wang J. (2021). Smart materials for microrobots. Chem. Rev..

[28] Martínez-Pedrero F., González-Banciella A., Camino A., Mateos-Maroto A., Ortega F., Rubio R.G., Pagonabarraga I., Calero C. (2021). Static and Dynamic Self-Assembly of Pearl-Like-Chains of Magnetic Colloids Confined at Fluid Interfaces. Small.

[29] Lu X., Wei Y., Ou H., Zhao C., Shi L., Liu W. (2021). Universal Control for Micromotor Swarms with a Hybrid Sonoelectrode. Small.

[30] Zhang J., Mou F., Wu Z., Song J., Kauffman J.E., Sen A., Guan J. (2021). Cooperative transport by flocking phototactic micromotors. Nanoscale Adv..

[31] Tong J., Wang D., Liu Y., Lou X., Jiang J., Dong B., Dong R., Yang M. (2021). Bioinspired micro/nanomotor with visible light energy–dependent forward, reverse, reciprocating, and spinning schooling motion. Proc. Natl. Acad. Sci. USA.

[32] Kopitca A., Latifi K., Zhou Q. (2021). Programmable assembly of particles on a Chladni plate. Sci. Adv..

[33] Wu X., Xue X., Wang J., Liu H. (2020). Phototropic Aggregation and Light Guided Long-Distance Collective Transport of Colloidal Particles. Langmuir.

[34] Wang Q., Yang L., Zhang L. (2021). Micromanipulation using reconfigurable self-assembled magnetic droplets with needle guidance. IEEE Trans. Autom. Sci. Eng..

[35] Wang X., Hu C., Schurz L., De Marco C., Chen X., Pané S., Nelson B.J. (2018). Surface-chemistry-mediated control of individual magnetic helical microswimmers in a swarm. ACS Nano.

[36] Kokot G., Sokolov A., Snezhko A. (2020). Guided Self-Assembly and Control of Vortices in Ensembles of Active Magnetic Rollers. Langmuir.

[37] Wang Q., Yang L., Wang B., Yu E., Yu J., Zhang L. (2018). Collective Behavior of Reconfigurable Magnetic Droplets *Via* Dyn. Self-Assem. ACS Appl. Mater. Interfaces.

[38] Cheng R., Huang W., Huang L., Yang B., Mao L., Jin K., ZhuGe Q., Zhao Y. (2014). Acceleration of Tissue Plasminogen Activator-Mediated Thrombolysis by Magnetically Powered Nanomotors. ACS Nano.

[39] Hu J., Huang S., Zhu L., Huang W., Zhao Y., Jin K., ZhuGe Q. (2018). Tissue Plasminogen Activator-Porous Magnetic Microrods for Targeted Thrombolytic Therapy after Ischemic Stroke. ACS Appl. Mater. Interfaces.

[40] Wang Q., Zhang L. (2020). Ultrasound Imaging and Tracking of Micro/Nanorobots: From Individual to Collectives. IEEE Open J. Nanotechnol..

[41] Wang Q., Tian Y., Du X., Ko H., Ip B.Y.M., Leung T.W.H., Yu S.C.H., Zhang L. (2022). Magnetic Navigation of Collective Cell Microrobots in Blood Under Ultrasound Doppler Imaging. IEEE/ASME Trans. Mechatron..

[42] Joh H., Fan D.E. (2021). Materials and Schemes of Multimodal Reconfigurable Micro/Nanomachines and Robots: Review and Perspective. Adv. Mater..

[43] Wang Q., Yu J., Yuan K., Yang L., Jin D., Zhang L. (2020). Disassembly and spreading of magnetic nanoparticle clusters on uneven surfaces. Appl. Mater. Today.

[44] Lin Z., Si T., Wu Z., Gao C., Lin X., He Q. (2017). Light-Activated Active Colloid Ribbons. Angew. Chem. Int. Ed..

[45] Wang Q., Yang L., Yu J., Chiu P.W.Y., Zheng Y.P., Zhang L. (2020). Real-Time Magnetic Navigation of a Rotating Colloidal Microswarm under Ultrasound Guidance. IEEE Trans. Biomed. Eng..

[46] Aziz A., Pane S., Iacovacci V., Koukourakis N., Czarske J., Menciassi A., Medina-Sánchez M., Schmidt O.G. (2020). Medical Imaging of Microrobots: Toward *Vivo* Appl. ACS Nano.

[47] Vilela D., Cossío U., Parmar J., Martínez-Villacorta A.M., Gómez-Vallejo V., Llop J., Sánchez S. (2018). Medical Imaging for the Tracking of Micromotors. ACS Nano.

[48] Yu J., Wang Q., Li M., Liu C., Wang L., Xu T., Zhang L. (2019). Characterizing Nanoparticle Swarms with Tuneable Concentrations for Enhanced Imaging Contrast. IEEE Robot. Autom. Lett..

[49] Wang Q., Yang S., Zhang L. (2022). Magnetic Actuation of a Dynamically Reconfigurable Microswarm for Enhanced Ultrasound Imaging Contrast. IEEE/ASME Trans. Mechatron..

[50] Yu J., Yang L., Zhang L. (2018). Pattern Generation and Motion Control of a Vortex-Like Paramagnetic Nanoparticle Swarm. Int. J. Robot. Res..

[51] Massana-Cid H., Meng F., Matsunaga D., Golestanian R., Tierno P. (2019). Tunable Self-Healing of Magnetically Propelling Colloidal Carpets. Nat. Commun..

[52] Dong X., Sitti M. (2020). Controlling Two-Dimensional Collective Formation and Cooperative Behavior of Magnetic Microrobot Swarms. Int. J. Robot. Res..

[53] Hernàndez-Navarro S., Tierno P., Farrera J.A., Ignés-Mullol J., Sagués F. (2014). Reconfigurable Swarms of Nematic Colloids Controlled by Photoactivated Surface Patterns. Angew. Chem. Int. Ed..

[54] Singh D.P., Choudhury U., Fischer P., Mark A.G. (2017). Non-Equilibrium Assembly of Light-Activated Colloidal Mixtures. Adv. Mater..

[55] Aubret A., Youssef M., Sacanna S., Palacci J. (2018). Targeted assembly and synchronization of self-spinning microgears. Nat. Phys..

[56] Li Z., Zhang H., Wang D., Gao C., Sun M., Wu Z., He Q. (2020). Reconfigurable Assembly of Active Liquid Metal Colloidal Cluster. Angew. Chem..

[57] Xu T., Soto F., Gao W., Dong R., Garcia-Gradilla V., Magaña E., Zhang X., Wang J. (2015). Reversible Swarming and Separation of Self-Propelled Chemically Powered Nanomotors under Acoustic Fields. J. Am. Chem. Soc..

[58] Ma F., Wang S., Wu D.T., Wu N. (2015). Electric-Field-Induced Assembly and Propulsion of Chiral Colloidal Clusters. Proc. Natl. Acad. Sci. USA.

[59] Yan J., Han M., Zhang J., Xu C., Luijten E., Granick S. (2016). Reconfiguring Active Particles by Electrostatic Imbalance. Nat. Mater..

[60] Ahmed D., Baasch T., Blondel N., Läubli N., Dual J., Nelson B.J. (2017). Neutrophil-Inspired Propulsion in a Combined Acoustic and Magnetic Field. Nat. Commun..

[61] Tang S., Zhang F., Zhao J., Talaat W., Soto F., Karshalev E., Chen C., Hu Z., Lu X., Li J. (2019). Structure-Dependent Optical Modulation of Propulsion and Collective Behavior of Acoustic/Light-Driven Hybrid Microbowls. Adv. Funct. Mater..

[62] Ji F., Jin D., Wang B., Zhang L. (2020). Light-driven hovering of a magnetic microswarm in fluid. ACS Nano.

[63] Abbott J.J., Diller E., Petruska A.J. (2020). Magnetic methods in robotics. Annu. Rev. Control. Robot. Auton. Syst..

[64] Yang S., Wang Q., Jin D., Du X., Zhang L. (2022). Probing Fast Transformation of Magnetic Colloidal Microswarms in Complex Fluids. ACS Nano.

[65] Yu J., Wang B., Du X., Wang Q., Zhang L. (2018). Ultra-Extensible Ribbon-Like Magnetic Microswarm. Nat. Commun..

[66] Jin D., Yu J., Yuan K., Zhang L. (2019). Mimicking the Structure and Function of Ant Bridges in a Reconfigurable Microswarm for Electronic Applications. ACS Nano.

[67] Yu J., Xu T., Lu Z., Vong C.I., Zhang L. (2017). On-demand disassembly of paramagnetic nanoparticle chains for microrobotic cargo delivery. IEEE Trans. Robot..

[68] van Reenen A., de Jong A.M., Prins M.W. (2015). Transportation, dispersion and ordering of dense colloidal assemblies by magnetic interfacial rotaphoresis. Lab Chip.

[69] Yigit B., Alapan Y., Sitti M. (2020). Cohesive Self-Organization of Mobile Microrobotic Swarms. Soft Matter.

[70] Yigit B., Alapan Y., Sitti M. (2019). Programmable Collective Behavior in Dynamically Self-Assembled Mobile Microrobotic Swarms. Adv. Sci..

[71] Martinez-Pedrero F., Tierno P. (2015). Magnetic Propulsion of Self-Assembled Colloidal Carpets: Efficient Cargo Transport *Via* A Conveyor-Belt Eff. Phys. Rev. Appl..

[72] Snezhko A., Aranson I.S. (2011). Magnetic Manipulation of Self-Assembled Colloidal Asters. Nat. Mater..

[73] Snezhko A., Belkin M., Aranson I., Kwok W.K. (2009). Self-Assembled Magnetic Surface Swimmers. Phys. Rev. Lett..

[74] Xie H., Sun M., Fan X., Lin Z., Chen W., Wang L., Dong L., He Q. (2019). Reconfigurable Magnetic Microrobot Swarm: Multimode Transformation, Locomotion, and Manipulation. Sci. Robot..

[75] Martinez-Pedrero F., Cebers A., Tierno P. (2016). Dipolar rings of microscopic ellipsoids: Magnetic manipulation and cell entrapment. Phys. Rev. Appl..

[76] Driscoll M., Delmotte B., Youssef M., Sacanna S., Donev A., Chaikin P. (2017). Unstable fronts and motile structures formed by microrollers. Nat. Phys..

[77] Ibele M., Mallouk T.E., Sen A. (2009). Schooling Behavior of Light-Powered Autonomous Micromotors in Water. Angew. Chem. Int. Ed..

[78] Liang X., Mou F., Huang Z., Zhang J., You M., Xu L., Luo M., Guan J. (2020). Hierarchical Microswarms with Leader-Follower-Like Structures: Electrohydrodynamic Self-Organization and Multimode Collective Photoresponses. Adv. Funct. Mater..

[79] Mou F., Zhang J., Wu Z., Du S., Zhang Z., Xu L., Guan J. (2019). Phototactic Flocking of Photochemical Micromotors. iScience.

[80] Mou F., Li X., Xie Q., Zhang J., Xiong K., Xu L., Guan J. (2019). Active Micromotor Systems Built from Passive Particles with Biomimetic Predator-Prey Interactions. ACS Nano.

[81] He W., Frueh J., Hu N., Liu L., Gai M., He Q. (2016). Guidable Thermophoretic Janus Micromotors Containing Gold Nanocolorifiers for Infrared Laser Assisted Tissue Welding. Adv. Sci..

[82] Bruus H. (2012). Acoustofluidics 7: The acoustic radiation force on small particles. Lab Chip.

[83] Doinikov A.A. (2001). Acoustic Radiation Interparticle Forces in a Compressible Fluid. J. Fluid Mech..

[84] Bricard A., Caussin J.B., Desreumaux N., Dauchot O., Bartolo D. (2013). Emergence of Macroscopic Directed Motion in Populations of Motile Colloids. Nature.

[85] Li J., Li T., Xu T., Kiristi M., Liu W., Wu Z., Wang J. (2015). Magneto-Acoustic Hybrid Nanomotor. Nano Lett..

[86] Kaynak M., Ozcelik A., Nourhani A., Lammert P.E., Crespi V.H., Huang T.J. (2017). Acoustic Actuation of Bioinspired Microswimmers. Lab Chip.

[87] Palacci J., Sacanna S., Steinberg A.P., Pine D.J., Chaikin P.M. (2013). Living Crystals of Light-Activated Colloidal Surfers. Science.

[88] Chen H., Wang Y., Liu Y., Zou Q., Yu J. (2022). Sensing of Fluidic Features Using Colloidal Microswarms. ACS Nano.

[89] Yu J., Jin D., Chan K.F., Wang Q., Yuan K., Zhang L. (2019). Active Generation and Magnetic Actuation of Microrobotic Swarms in Bio-Fluids. Nat. Commun..

[90] Zhou H., Dong G., Gao G., Du R., Tang X., Ma Y., Li J. (2022). Hydrogel-Based Stimuli-Responsive Micromotors for Biomedicine. Cyborg Bionic Syst..

[91] Liang X., Chen Z., Deng Y., Liu D., Liu X., Huang Q., Arai T. (2023). Field-controlled microrobots fabricated by photopolymerization. Cyborg Bionic Syst..

[92] Wu Z., Chen Y., Mukasa D., Pak O.S., Gao W. (2020). Medical Micro/Nanorobots in Complex Media. Chem. Soc. Rev..

[93] Wang Q., Xiang N., Lang J., Wang B., Jin D., Zhang L. (2023). Reconfigurable Liquid-Bodied Miniature Machines: Magnetic Control and Microrobotic Applications. Adv. Intell. Syst..

[94] Wang Q., Chan K.F., Schweizer K., Du X., Jin D., Yu S.C.H., Nelson B.J., Zhang L. (2021). Ultrasound Doppler-guided real-time navigation of a magnetic microswarm for active endovascular delivery. Sci. Adv..

[95] Ahmed D., Sukhov A., Hauri D., Rodrigue D., Maranta G., Harting J., Nelson B.J. (2021). Bioinspired acousto-magnetic microswarm robots with upstream motility. Nat. Mach. Intell..

[96] Sitti M., Wiersma D.S. (2020). Pros and Cons: Magnetic *Versus* Opt. Microrobots. Adv. Mater..

[97] Wang B., Chan K.F., Yu J., Wang Q., Yang L., Chiu P.W.Y., Zhang L. (2018). Reconfigurable Swarms of Ferromagnetic Colloids for Enhanced Local Hyperthermia. Adv. Funct. Mater..

[98] Cheng X., Sun R., Yin L., Chai Z., Shi H., Gao M. (2017). Light-triggered assembly of gold nanoparticles for photothermal therapy and photoacoustic imaging of tumors in vivo. Adv. Mater..

[99] Higbee-Dempsey E., Amirshaghaghi A., Case M.J., Miller J., Busch T.M., Tsourkas A. (2019). Indocyanine green–coated gold nanoclusters for photoacoustic imaging and photothermal therapy. Adv. Ther..

[100] Seo S.H., Kim B.M., Joe A., Han H.W., Chen X., Cheng Z., Jang E.S. (2014). NIR-light-induced surface-enhanced Raman scattering for detection and photothermal/photodynamic therapy of cancer cells using methylene blue-embedded gold nanorod@ SiO2 nanocomposites. Biomaterials.

[101] Wang W., Duan W., Zhang Z., Sun M., Sen A., Mallouk T.E. (2015). A tale of two forces: Simultaneous chemical and acoustic propulsion of bimetallic micromotors. Chem. Commun..

[102] Jin D., Yuan K., Du X., Wang Q., Wang S., Zhang L. (2021). Domino reaction encoded heterogeneous colloidal microswarm with on-demand morphological adaptability. Adv. Mater..

[103] Wang B., Liu D., Liao Y., Huang Y., Ni M., Wang M., Ma Z., Wu Z., Lu Y. (2022). Spatiotemporally Actuated Hydrogel by Magnetic Swarm Nanorobotics. ACS Nano.

[104] Zou Q., Du X., Liu Y., Chen H., Wang Y., Yu J. (2022). Dynamic Path Planning and Motion Control of Microrobotic Swarms for Mobile Target Tracking. IEEE Trans. Autom. Sci. Eng..

[105] Wang Q., Du X., Jin D., Zhang L. (2022). Real-Time Ultrasound Doppler Tracking and Autonomous Navigation of a Miniature Helical Robot for Accelerating Thrombolysis in Dynamic Blood Flow. ACS Nano.

[106] Schmidt C.K., Medina-Sánchez M., Edmondson R.J., Schmidt O.G. (2020). Engineering microrobots for targeted cancer therapies from a medical perspective. Nat. Commun..

[107] Lu J., Liu Y., Huang W., Bi K., Zhu Y., Fan Q. (2022). Robust Control Strategy of Gradient Magnetic Drive for Microrobots Based on Extended State Observer. Cyborg Bionic Syst..

[108] Wang Q., Jin D., Wang B., Xia N., Ko H., Ip B.Y.M., Leung T.W.H., Yu S.C.H., Zhang L. (2022). Reconfigurable magnetic microswarm for accelerating tPA-mediated thrombolysis under ultrasound imaging. IEEE/ASME Trans. Mechatron..

